# Premature neonatal gut microbial community patterns supporting an epithelial TLR-mediated pathway for necrotizing enterocolitis

**DOI:** 10.1186/s12866-021-02285-0

**Published:** 2021-08-06

**Authors:** Alexander G. Shaw, Kathleen Sim, Graham Rose, David J. Wooldridge, Ming-Shi Li, Raju V. Misra, Saheer Gharbia, J. Simon Kroll

**Affiliations:** 1 Department of Infectious Disease Epidemiology, Imperial College London, Wright-Fleming Institute, Praed Street, London, W2 1PG UK; 2 Department of Medicine, Section of Paediatrics, Imperial College London, London, W2 1PG UK; 3 Genomic Research Unit, Public Health England, Microbiology Services, 61 Colindale Avenue, London, NW9 5EQ UK; 4 Molecular Biology Laboratories, The Natural History Museum, Cromwell Road, London, SW7 5BD UK

**Keywords:** Metagenome, Necrotising enterocolitis, Premature infant, Microbiome, TLR4, TLR9

## Abstract

**Background:**

Necrotising enterocolitis (NEC) is a devastating bowel disease, primarily affecting premature infants, with a poorly understood aetiology. Prior studies have found associations in different cases with an overabundance of particular elements of the faecal microbiota (in particular Enterobacteriaceae or *Clostridium perfringens*), but there has been no explanation for the different results found in different cohorts. Immunological studies have indicated that stimulation of the TLR4 receptor is involved in development of NEC, with TLR4 signalling being antagonised by the activated TLR9 receptor. We speculated that differential stimulation of these two components of the signalling pathway by different microbiota might explain the dichotomous findings of microbiota-centered NEC studies. Here we used shotgun metagenomic sequencing and qPCR to characterise the faecal microbiota community of infants prior to NEC onset and in a set of matched controls. Bayesian regression was used to segregate cases from control samples using both microbial and clinical data.

**Results:**

We found that the infants suffering from NEC fell into two groups based on their microbiota; one with low levels of CpG DNA in bacterial genomes and the other with high abundances of organisms expressing LPS. The identification of these characteristic communities was reproduced using an external metagenomic validation dataset. We propose that these two patterns represent the stimulation of a common pathway at extremes; the LPS-enriched microbiome suggesting overstimulation of TLR4, whilst a microbial community with low levels of CpG DNA suggests reduction of the counterbalance to TLR4 overstimulation.

**Conclusions:**

The identified microbial community patterns support the concept of NEC resulting from TLR-mediated pathways. Identification of these signals suggests characteristics of the gastrointestinal microbial community to be avoided to prevent NEC. Potential pre- or pro-biotic treatments may be designed to optimise TLR signalling.

**Supplementary Information:**

The online version contains supplementary material available at 10.1186/s12866-021-02285-0.

## Background

### Necrotising enterocolitis

Necrotising enterocolitis (NEC) is a devastating bowel disease primarily affecting premature infants, the aetiology of which is poorly understood. The increasing incidence of the disease which has come with the advent of improved neonatal intensive care, and our ability to keep more and more premature infants alive, combined with the high mortality rate, limited treatment options, rapid onset, and lack of a screening tool, has made research into the disease a priority.

Bacteria are considered to play a key role in the pathogenesis of NEC – the condition does not occur in sterile settings (e.g. in utero or in germ-free animal models). Antibiotics are used to treat the disease, but paradoxically, antibiotic treatment early in life as empirical therapy for sepsis been shown to increase the risk of developing the disease [1].

We have previously described our findings of two different microbial signatures anticipating the development of NEC in a cohort of premature infants [2]. Using 16S rRNA characterisation of faecal samples collected from the infants from birth until disease onset, we showed that there was either a ‘bloom’ of *C. perfringens* immediately prior to diagnosis, or, unusually high levels of Enterobacteriaceae from birth which persisted until NEC developed. Other investigators have reported similar findings [3–5], posing a question as to how such very different organisms can provoke the same comparable pathogenic process.

### The role of TLR4 and 9 in necrotising enterocolitis

In a mouse model of NEC, the pattern recognition receptor TLR4, a key component of the innate immune system, has been found to play a central role, its deletion being protective [6]. TLR4 expression in the fetal/neonatal murine gut is temporally regulated, levels in the gut epithelium and endothelium rising as the foetus matures and then rapidly falling in the perinatal period [7]. On premature (over)exposure of neonatal mouse epithelial TLR4 to its ligand (lipopolysaccharide (LPS): a major component of the outer membrane of Gram-negative bacteria), binding leads to activation of the NF-KB pathway and destruction of the gut epithelium that is the hallmark of NEC [7].

A similar pattern of TLR4 expression has been reported in human infants, with a peak at 16 weeks gestation and greatly increased expression at 21 weeks gestation compared to 4 months after birth [8]. An infant born prematurely could therefore be expected to have a comparatively high expression of TLR4 in the gut epithelium, and premature exposure to a microbiota by mischance dominated by Gram-negative organisms, leading to widespread activation of TLR4, to carry the risk of catastrophic epithelial damage.

Of course, not all infants colonised with high levels of Gram-negative organisms develop NEC. TLR4 signalling is down-regulated by TLR9, for which the activating ligand is the CpG motif in DNA [9]. CpG motifs are more prevalent in bacterial than in eukaryotic DNA, although the frequency/kb varies greatly across the bacterial kingdom [10]. Activation of TLR9 acts to inhibit TLR4 mediated signalling via IL-1R-asscoaited kinase M [7]. TLR9 therefore acts to prevent a constant inflammatory response to the commensal bacteria that are resident in the lumen of the gut [11].

We hypothesised that an imbalance in the stimulation of TLR4 and TLR9 in the faecal gut, potentially leading to the development of NEC, could be observed in part through calculation of the abundance of Gram-negative bacteria and bacterial DNA CpG content which would serve as proxies of immuno-stimulation. To address this, we undertook both a metagenomic analysis and a bacterial quantification by qPCR of the microbial content of faecal samples closest to the time of diagnosis in infants prior to the development of NEC, and in a set of paired controls. From this we calculated the number of CpG motifs present per gram of faecal matter. Quantitative PCR data and taxonomic identifications were also used to estimate the number of Gram-negative bacteria present. We report here that faecal samples collected from infants taken just before NEC development have either a lower CpG motif frequency in bacterial DNA or a higher amount of Gram-negative bacteria per gram of faeces compared to samples from control infants matched for postnatal age. These findings were validated using an external dataset which also included both a metagenomic analysis and bacterial quantification for faecal samples collected from premature infants.

## Results

### Sequencing results

**Fig. 1:**
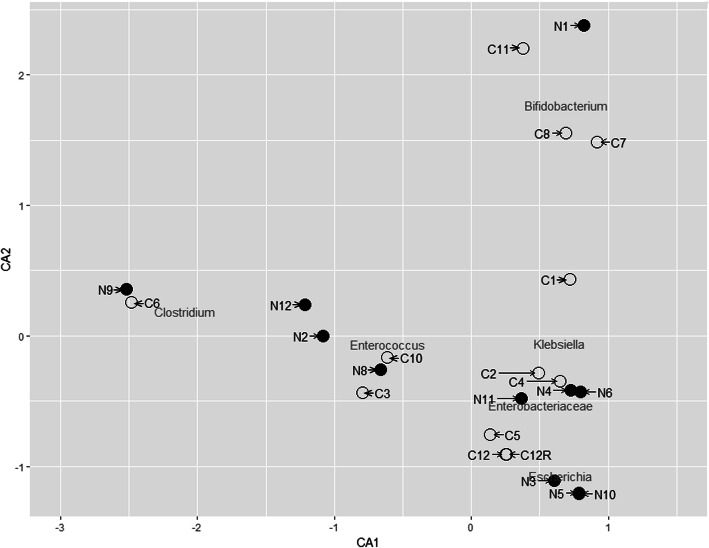
Canonical correlation analysis plot showing the similarity of the processed samples. Analysis was performed using data quantifying the most abundant taxonomic groups comprising 95% of the sequencing reads (*n* = 24; 11 NEC samples (black), 11 control samples (white), 1 technical repeat (white, C12R)). Taxonomic names indicate the major bacterial groups associated with nearby samples which drive the separation

Twenty-six samples entered the shotgun metagenomic sequencing pipeline. These comprised 12 NEC samples (N1-N12) collected as close to diagnosis as possible, 12 matched control samples (C1-C12), a DNA extraction negative control and a technical repeat of sample C12. The negative control and two samples, N7 and C9, failed library amplification (requiring more than eight PCR cycles). Canonical correlation analysis (CCA) was used to compare the similarity of samples by microbial content, confirming that the technical repeat clustered tightly to its pair (see Fig. 1), indicating the robustness of the sequencing pipeline.

### Microbial communities of sequenced samples

**Fig. 2:**
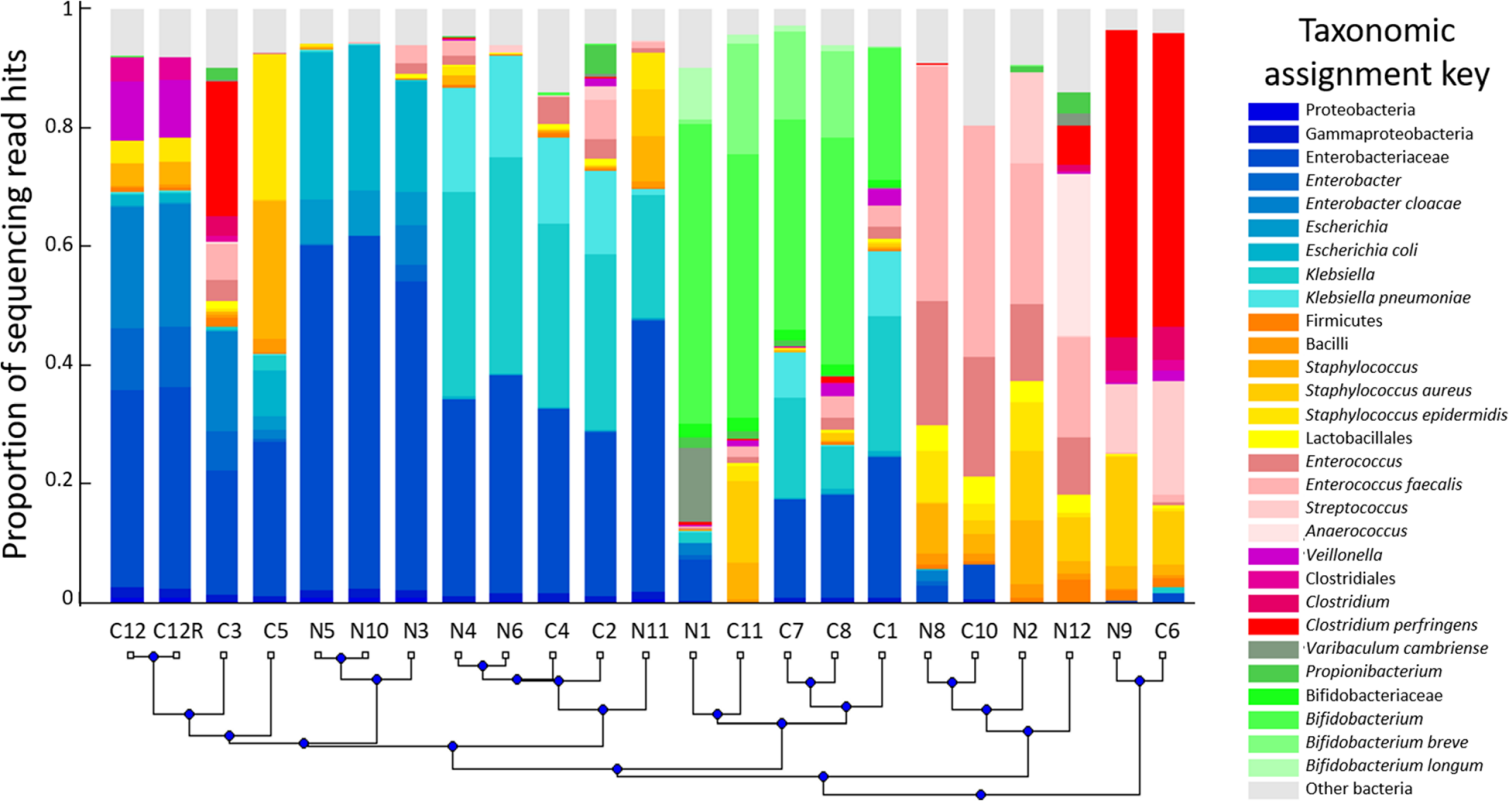
Samples clustered by taxonomic assignment of sequencing reads. Y axis indicates proportion of reads assigned to each taxonomic category as coded in the colour key. The categories are spread over multiple taxonomic levels, with reads binned according to highest resolution possible. X axis indicates the samples, clustered by similarity (*n* = 22; 11 NEC samples, 11 control samples)

After removal of failed samples, controls and repeats, eleven NEC samples and eleven control samples remained, of which twenty were matched pairs. Samples were clustered according to the similarity of their microbial communities (see Fig. 2), displaying some loose grouping between sample types. NEC samples tended towards domination by either Enterobacteriaceae or *Enterococcus*, *Staphylococcus*, *Anaerococcus* or *Clostridium* species. Some control samples fell into these categories, whilst others featured high abundances of Bifidobactericeae.

### Quantification of the bacterial communities

qPCR data for each faecal sample was corrected for the 16S rRNA gene copy number of its bacterial community. This provided an estimate of the number of bacteria per gram of faeces (see Additional file 1). No significant difference in bacterial abundance was found between control and NEC samples.

### TLR9 stimulatory potential of typical premature infant gut colonisers

**Fig. 3:**
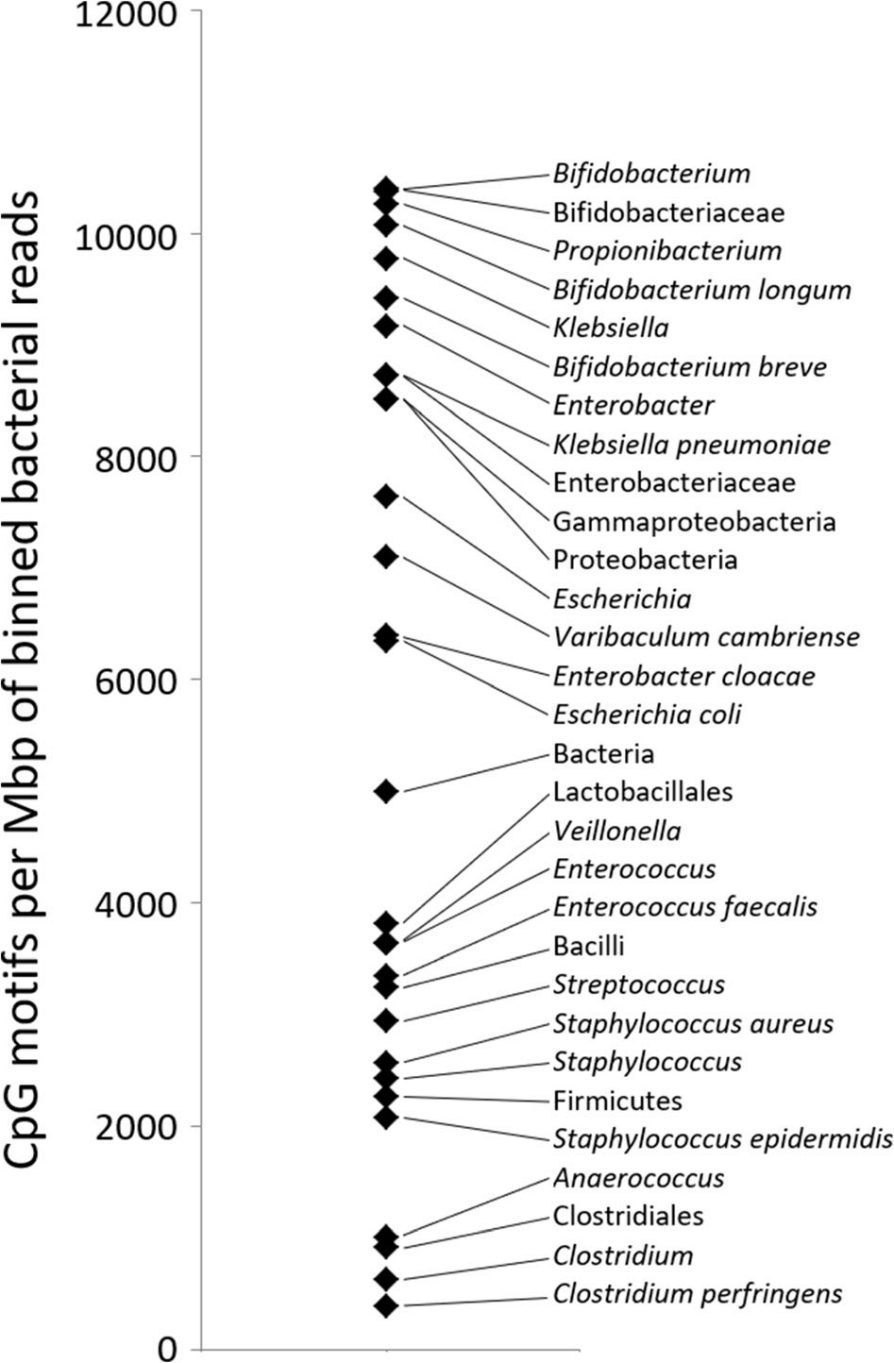
CpG motif frequency in premature infant gut colonisers. Binned bacterial reads for the taxonomic groups making up the top 95% of classifications are stratified according to the number of CpG motifs per megabase of DNA

We sought to establish the stimulatory properties of the bacteria present in the guts of premature infants with regard to TLR9 through calculation of the frequency of CpG DNA per megabase of their sequenced DNA. Taxonomic groups relating to *Bifidobacterium* had the highest frequency of CpG motifs, whilst *Clostridium*, *Anaerococcus* and *Staphylococcus* taxonomic groups had a CpG motif frequency between 4 and 25% of this (see Fig. 3). These results are consistent with previous findings [10].

### Stratification of samples by immunostimulatory proxies

Following prior studies in neonatal mice, [6, 7] we hypothesised that infants at high risk of NEC would either display over-stimulation of TLR4 or under stimulation of TLR9. To investigate whether the segregated into the two categories we first sought to identify the infants most likely to fall into either category.

**Fig. 4:**
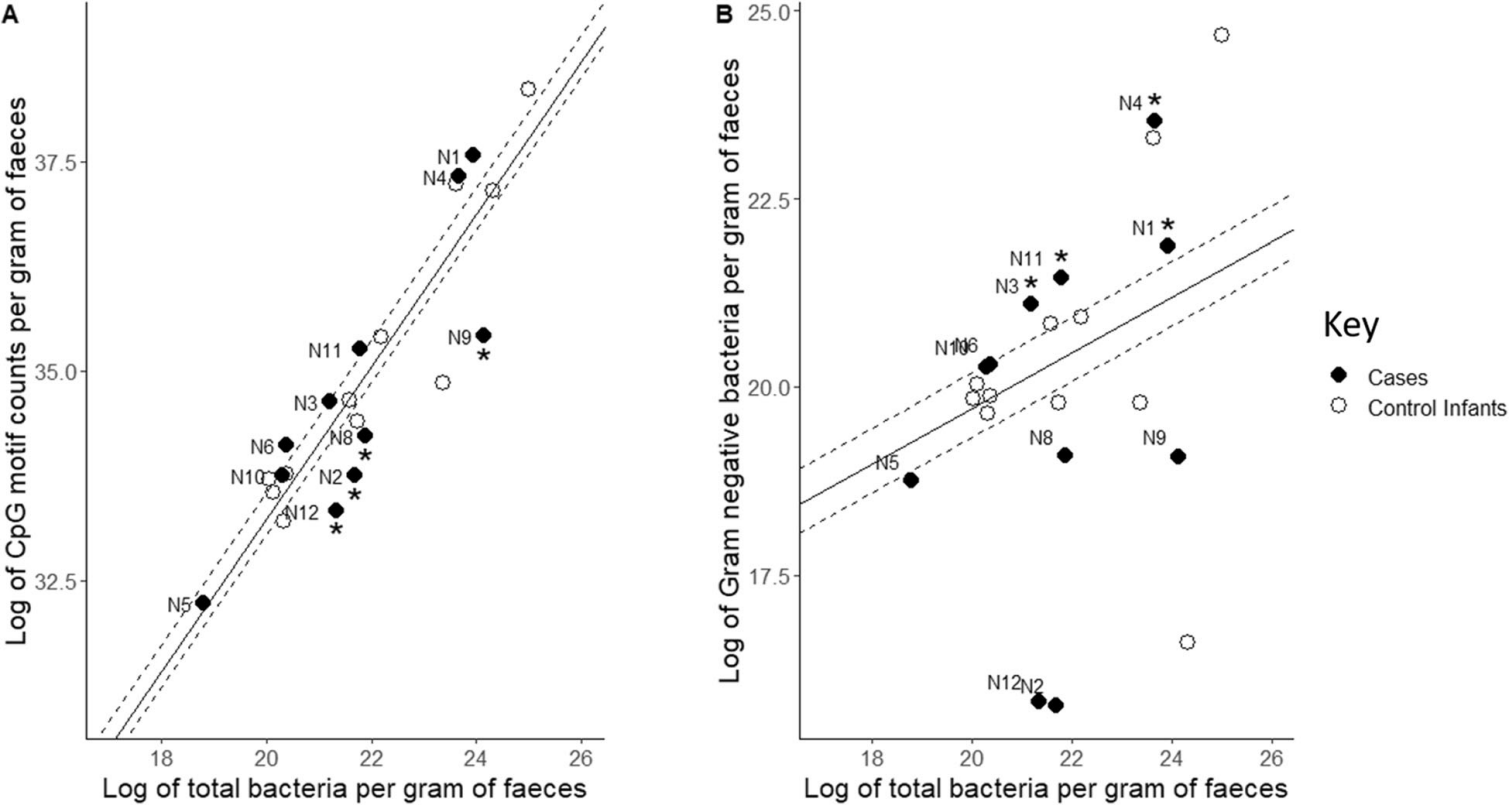
Stratification of samples by bacterial load and **A** CpG DNA content or **B** Gram-negative bacteria. Control and NEC samples are stratified by bacteria per gram of faeces (x axis) and **A** the occurrences of CpG DNA per gram of faeces (y axis) and **B** number of Gram-negative bacteria per gram of faeces (y axis). The solid diagonal lines indicate the relationship established between each pair of factors for control infants using linear regression. The interquartile range (IQR) of the deviation of control samples from the regression line is indicated by dashed lines. Stars indicate NEC cases identified as either having particularly low CpG DNA per gram of faeces or particularly high abundances of Gram-negative bacteria compared to control infants, as defined by being outside the IQR of the control samples

To quantify the overall immunostimulatory potential of the faecal microbiota for TLR 9, we estimated the abundance of CpG DNA per gram of faeces and the total number of bacteria per gram of faeces. The expected relationship between the two would be linear; an increase in the amount of bacteria would lead to a proportional increase in CpG motifs. The majority of samples indeed followed this relationship (see Fig. 4A). However, four NEC cases (N2, N8, N9 and N12) were found to have a particularly low ratio of CpG motifs to bacteria.

We then sought to identify the NEC samples most likely to feature high immunostimulation of TLR 4 due to an overabundance of its ligand, LPS. As a proxy for the abundance of LPS, the number of Gram-negative bacteria per gram of faeces was calculated by classifying the previously quantified bacterial taxonomic groups according to Gram staining (see Fig. 4B). Four NEC cases (N1, N3, N4, N11) displayed an over-abundance of Gram-negative bacteria compared to the control samples.

The remaining three NEC cases (N5, N6, N10) were not assigned an initial classification.

### Stratification of samples by immunostimulatory proxies and clinical factors

Two Bayesian linear regression models were used to segregate the two groups of classified NEC cases from the control infants, with each model focusing on one of the immunostimulatory proxies. In anticipation that the abundance of these proxies and the bacteria per gram of faeces would not remain static over time, day of life and gestation at birth were included as additional factors along with days of antibiotics prior to sampling. Reclassification of the NEC cases was permitted in response to the inclusion of these additional factors, with the overall aim of achieving the most accurate segregation of cases and controls.

**Fig. 5:**
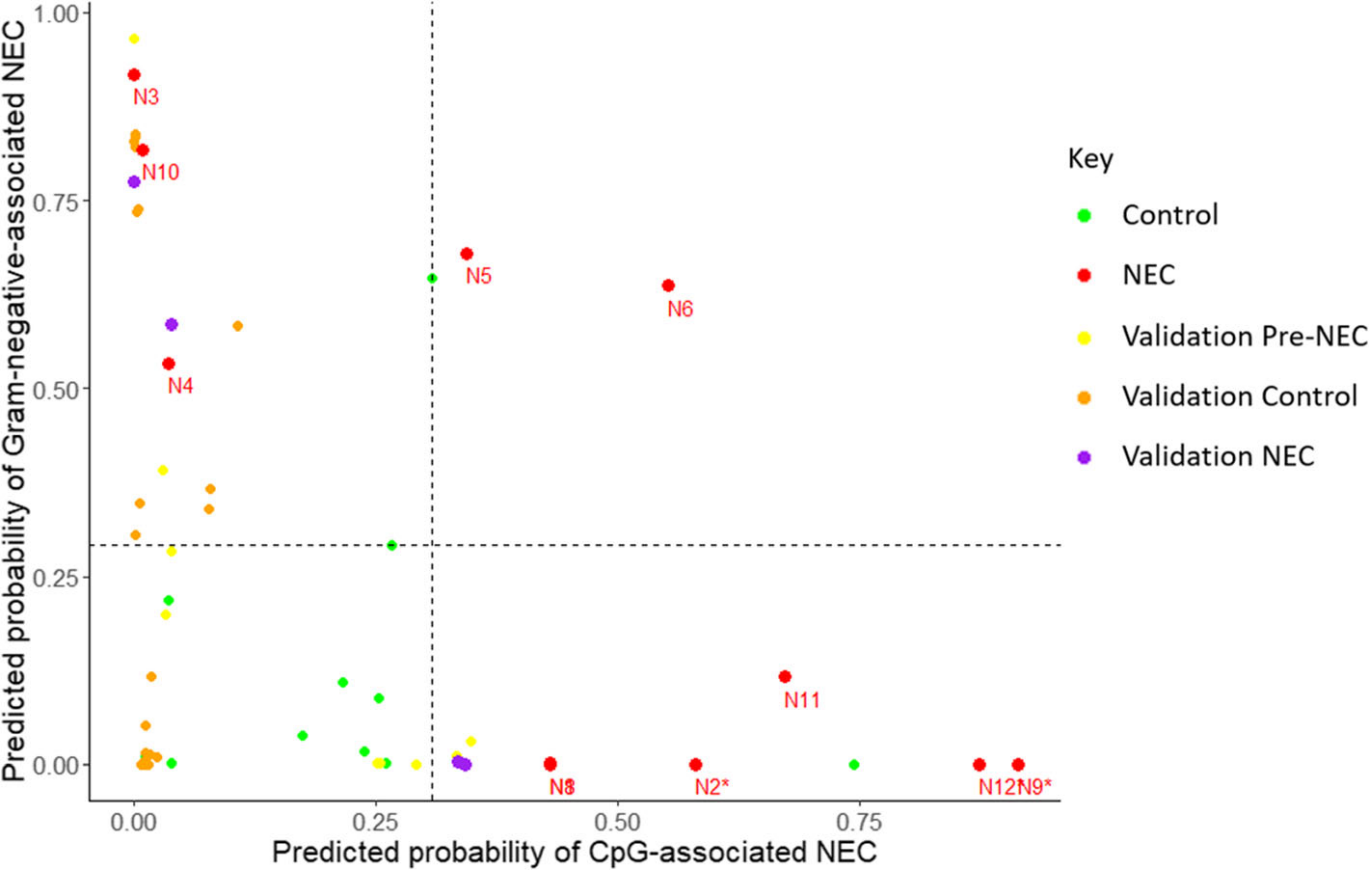
Predicted probabilities from the Bayesian regression models for training and validation datasets. Predicted probabilities for the CpG-related NEC and Gram-negative-related NEC models are shown for 11 control infants, 11 NEC cases and for the validation dataset. Dashed lines indicate the 90% quantile of predicted probability for the eleven control samples along each axis. “NEC” samples are the closest available samples to an infant’s NEC diagnosis for each dataset and “Pre-NEC” refers to any prior samples from these infants. NEC cases that received no antibiotics prior to samples being taken are marked with stars. The points for samples “N1” and “N8” are overlaid

The first model, aiming to identify the risk of NEC associated with low stimulation of TLR9 (“CpG-associated NEC”), sought to initially separate the cases highlighted in Fig. 4A) from control infants. Predicted probabilities of NEC risk were calculated for each NEC and control sample. Seven cases had predicted probabilities of NEC greater than the 90% quantile of control infant risk probabilities (see Fig. 5). Increased risk was associated with lower CpG motifs per bacteria within the sample, a lower total abundance of bacteria, fewer days with antibiotics, increased days of life and decreased gestation at birth.

The second model, aiming to identify the risk of NEC associated with over-stimulation of TLR4 (“Gram-negative-associated NEC”) sought to initially segregate the cases highlighted in Fig. 4B) from control infants. Five cases had predicted probabilities of NEC greater than the 90% quantile of control infant risk probabilities (see Fig. 5). Increased risk for Gram-negative-associated NEC was associated with increased proportion of the bacterial community being Gram-negative, increased overall bacterial abundance, increased days of antibiotics, increased day of life and increased gestation at birth. Gram-negative bacteria comprised 93% of this cohort’s total bacteria, compared to only 51% in the control infants. The coefficients of the two models are shown in additional file 2. NEC severity was not associated with predicted probabilities.

The two categories of cases were clearly separated by antibiotics use; of the three cases predicted high risk exclusively by the Gram-negative-associated NEC model all had antibiotics courses prior to NEC (a mean of 9 days). The six cases with predicted high risk exclusively of CpG-associated NEC had received a mean of only 1 day of antibiotics, with three cases having received none.

To test whether these immunostimulatory signatures were reproducible, an external metagenomic dataset with quantification by digital droplet PCR was used for validation [12]. Samples from five control infants and three infants that developed NEC were classified using the models. One of the cases developed NEC twice, and each incident was included. The cases each had a sample within a five-day window prior to NEC diagnosis, and a total of nine earlier samples. Twenty-three samples were tested from the five control infants. Sequencing data and quantification of CpG and bacteria were processed using the same pipeline as our own dataset. These data were entered into the two models with the coefficients derived from the testing on our own cohort to give predictive probabilities (See Fig. 5).

The samples closest to NEC diagnosis from one case and the sample prior to the first incidence of NEC from the recurrent case had high predicted probabilities of CpG-associated NEC along with two samples taken 2 and 6 days prior to the sample closest to NEC. The remaining NEC case and the second incidence of the recurrent case of NEC (following a ten-day treatment with antibiotics) had a high predicted probability of Gram-negative associated NEC, along with two samples taken 1 and 2 days prior. Seven validation control samples had high risk of Gram-negative associated NEC and all were within the first 2 weeks of life, whilst samples from NEC cases attributed high risk were all from week three to week eight of life.

## Discussion

In this study we have used characterisation of the infant faecal microbiota by shotgun metagenomics and qPCR to demonstrate how infants who go on to develop NEC display characteristic communities of faecal microbiota prior to diagnosis. These communities feature high proportions of LPS-expressing bacteria and/or a low frequency of CpG motifs within the bacterial DNA. These findings have been reproduced in an external cohort and fall in line with a recent theory concerning the development of NEC; we observe a community with either high levels of LPS that could stimulate the TLR4 receptor leading to inflammation, or low CpG frequencies which could lead to reduced TLR9 signalling and reduced IRAK-M dependent inhibition of TLR4 [7].

The mostly dichotomous nature of our findings suggests why prior microbial studies have confusingly implicated a range of organisms, with some studies highlighting association with an excess of Enterobacteriaceae [2, 3, 5, 13], whilst others with a range of organisms including *Clostridium* and *Staphylococcus* species [2, 4, 13]. We suggest that where NEC is associated with an Enterobacteriaceae-dominated microbiota the pathological basis for the epithelial necrosis is overstimulation of TLR4 by over-abundant LPS, while where the association is with the bloom of a pathogen like *C. perfringens*, it is the (strikingly) low CpG frequency in the resulting microbiota that leads to a failure of counter-regulation of TLR4 through TLR9.

Both of our models include terms representing temporal as well as bacterial associations, as the occurrence of the microbial patterns must be considered in relation to clinical time-courses. In each model, increase in risk is associated with increased day of life, in line with the recognised paucity of NEC cases prior to 8–10 days post-partum [14]. For our CpG-associated NEC model this association may represent the requirement that the gut becomes anaerobic prior to the flourishing of *Clostridium* species - the organisms with the lowest CpG motifs observed within our cohort. For the Gram-negative-associated NEC model increased risk associated with increased day of life may reflect the uncharacteristic persistence of Gram-negative bacteria within infant’s gut microbiota. A shift away from this pattern is typically seen from week three of life as the gut becomes more anaerobic and obligate anaerobes begin to dominate [15]. Both in our study cohort and in the validation dataset there are a small number of early life control and pre-NEC samples - predominantly taken in the first 2 weeks of life - that have high levels of Gram-negative bacteria. Control samples collected after this time period tend not display this, with communities following the typical pattern of succession. However, in infants that develop NEC, the dominance of Gram-negative bacteria persists. Reduced gestation is also associated with increased risk of Gram-negative associated NEC, and this may relate to expression of TLR4 in the infant gut with gestational age, which is observed in mice to peak prior to birth [7]. These two factors combined could lead to high-risk infants experiencing the confluence of peaking TLR4 expression and prolonged exposure to high abundances of LPS.

LPS from different organisms causes varying degrees of TLR4 stimulation, with *Veillonella parvula* at one extreme causing minimal stimulation. This may be a characteristic of the Negativicutes class as a whole, given their evolutionary distance from other Gram-negative organisms, and may explain why Negativicutes have been negatively associated with NEC [5]. Further characterisation of the immune-stimulatory properties of individual Gram-negative bacterial species will be important to fully parametrise our Gram-negative-associated NEC model.

Our CpG-associated-NEC model identified an association between fewer days of antibiotic treatment and the development of NEC. While previous studies have shown that increased antibiotic usage is linked with NEC [1], in the case of these specific infants in our cohort, the reduced antibiotic duration may have facilitated the succession of the gut microbiota towards organisms such as Clostridia. In term and pre-term infants, antibiotic treatment has been demonstrated to lead to a higher proportion of Proteobacteria in the post-treatment gut microbiota [16, 17], as seen by the positive association between days of antibiotics and increased Gram-negative associated NEC risk. Interestingly, the validation case with two incidents of NEC was initially found at high risk of CpG-related NEC and then progressed to high risk of Gram-negative-associated NEC after an additional 10 days of antibiotic treatment. The proportion of the bacterial population that was Gram-negative was only 24% prior to the first incidence of NEC and had risen to 74% before the second incident.

Whilst our two models classify all eleven NEC cases in our cohort and the three validation cases as being at high risk, the credible intervals for the coefficients that were determined are wide and require further data to provide greater certainty. Both day of life and gestation were retained in the models given the highly time dependent nature of the remaining factors. The resulting coefficients were relatively small however, possibly due to the correlation with the other factors, for example the negative association between Gram negative bacteria in the faecal microbiota and day of life. Additional data points for the first 2 weeks of life or longitudinal data could better inform this relationship to avoid high risk classifications for healthy infants who lack long-term faecal colonisation by Gram-negative bacteria. We also acknowledge that whilst faeces is a convenient sampling methods, it may not contain a true representation of the gut microbiota at the site of the small intestine where NEC commonly occurs [18]. Whilst our method of bacterial quantification includes a correction for 16S rRNA gene copy number, the exact number of copies can vary even within genera [19]. Where sequencing reads could not be binned by species this could therefore lead to deviations in exact bacterial abundance.

Bifidobacterium species have been shown to have varied effects on NEC risk, despite similar levels of CpG DNA, hence there are likely to be additional factors, potentially species-specific, that require consideration. These include the reduction of ileum inflammation and improvement of intestinal integrity by *B.bifidum* in neonatal NEC rat models [20] and the production of the anti-inflammatory molecule indole-3-lactic acid demonstrated by *B. infantis* [21], compared to the lack of effect for *B. breve* on either intestinal permeability or intestinal mucosal inflammation seen in the PiPS probiotics trial [22]. Further investigation to separate the influence of these factors would be of great importance to quantifying the value of Bifidobacterium species for NEC prevention, particularly with regard to the choice of species for potential probiotic treatments.

Although the predicted risk scores for the two models are plotted against each other in Fig. 5, the mathematical interaction between these two pathways is unknown. One control sample has relatively high risk by both models yet did not develop NEC. Mathematical characterisation of the interactions between the two pathways would be essential to combine the two models into a single unified predictor of NEC risk, and would likely also require the interplay of other immunological meditators that could potentially be involved such as those discussed by Cho et al [23]*.*

## Conclusions

The microbial community patterns presented here support the possibility of NEC occurring due to TLR-mediated pathways. Our findings suggest that clinical management of very premature infants to avoid NEC may benefit from careful steering of the faecal microbiota community to avoid the development of either of the two potentially destructive patterns. The prophylactic use of antibiotics in the NICU has been observed to lead to increased community domination by Enterobacteriaceae [16], leading to microbiota conforming to our high Gram-negative pattern. Whilst a suitably anaerobic gut environment may harbour low-CpG organisms such as *Clostridium perfringens,* characteristic of our second destructive pattern, appropriate nutrition, with consideration of the use of pre- or probiotics, may promote community development towards a more beneficial pattern, dominated by bacteria such as *Bifidobacterium*. Interventions aimed at rehabilitating the gut microbiota should be designed so as to avoid avoiding both negative community patterns. We believe our findings provide an explanation for the different, yet consistent, microbiota associated with NEC that have been reported by researchers around the world over the course of decades, improving understanding of the interplay between the host and the resident gastrointestinal microbiota which may indicate new strategies for the prevention of NEC.

## Methods

### Study population

Faecal samples analysed were from infants enrolled on the “Defining the Intestinal Microbiota in Premature Infants” study between January 2011 and December 2012 at an Imperial College Healthcare National Health Service Trust neonatal intensive care unit (NICU) (St Mary’s Hospital, Queen Charlotte’s and Chelsea Hospital). Recruited infants were born before 32 completed weeks of gestation, with 369 of the 388 eligible infants being recruited over the two-year study period.

The study was approved by West London Research Ethics Committee Two, United Kingdom (reference number 10/H0711/39). Parents gave written informed consent for their infant to participate in the study.

### Sample collection

Faecal samples were collected by nursing staff from diapers using a sterile spatula, placed in a sterile DNase-, RNase-free Eppendorf tube, stored at − 20 °C within 2 h of collection and stored at − 80 °C within 5 days. Almost every faecal sample produced by each enrolled infant between recruitment and discharge was collected.

### Case definition, control selection, and clinical management

NEC cases were defined using the Vermont Oxford Network criteria and staged using the Bell modified staging criteria [24, 25]. The closest available sample prior to the day of diagnosis was selected for twelve infants with NEC. For each of these samples, a sample from a control infant was matched by day of life the samples were taken, gestation at birth, delivery mode and days of any antibiotics use of each of the infants. Infant details are shown in Additional file 3. All infants received donor breast milk and/or mother’s breast milk, but no infant had begun breastfeeding prior to sample collection. Infants were not matched on feeds. Investigators were not involved in clinical care.

### DNA extraction and shotgun library preparation

DNA extractions and shotgun library preparation were performed as described previously [26], with the inclusion of a negative extraction control and a technical replicate (C12R). Briefly, DNA extractions were performed with 200 mg of faeces and the process included selective lysis of eukaryotic cells and degradation of eukaryotic DNA, followed by bead-beating to extract bacterial DNA prior to purification. The DNA was fragmented using the NEBNext dsDNA fragmentase kit (NEB), and shotgun libraries prepared using the KAPA HyperPrep kit (KAPA Biosystems). Ligated libraries were PCR amplified with the number of cycles being dependant on starting material (between two and eight cycles). The negative extraction control and two faceal samples (one NEC, one control) required > 8 PCR cycles, hence the samples were excluded from downstream analysis. The library was normalised, pooled and diluted to 1.6pM prior to loading on an Illumina NextSeq 500 system. The fragment size of the libraries sequenced ranged from 244 bp to 288 bp, with a mean of 261 bp.

### Shotgun metagenomic sequencing

Paired end sequencing was performed using a v2 300 cycle high output reagent kit (Illumina), generating over 300 million PE reads and yielding 90.4 Gbp of sequence data. Within the 22 infant samples, and excluding replicates, this translates to a mean 10.2 million PE reads or 2.6 Gbp sequence per sample (see Additional file 4).

### Sequencing data availability

Metagenomic sequencing data is available from the EBI European Nucleotide Archive under the study accession PRJEB24015.

### Processing of metagenomic sequences

Sequence quality was calculated using FastQC (v0.11.3) [27]. Read filtering was performed using Trimmomatic (v0.36) [28], with removal of the leading and trailing basepairs with phred qualities < 20 and reads with a mean base phred score quality < 20 over a 4 bp sliding window (parameters of: LEADING:20 TRAILING:20 SLIDINGWINDOW:4:20). Sequences with less than 40 bp remaining were discarded (MINLEN:40).

Surviving sequences were screened for human host and vector contamination using FastQ Screen (v0.8.0) and the short read aligner Bowtie2 (v2.2.6) [29, 30]. For validation samples, this screening process was performed on a subset of approximately 100,000 reads per sample. Reads were mapped against the human genome (GRCh38) and the UniVec (build 9.0) vector database, with unmapped reads continuing to the downstream analysis.

### Taxonomic identification and read characterisation

Taxonomic binning was performed using DIAMOND (v0.8.36) and MEGAN (v6.6.7) [31, 32]. A Lowest Common Ancestor algorithm with default MEGAN settings was used to assign an NCBI taxonomy, with relative abundances and binned bacterial reads being extracted. Taxonomic assignments were used to classify whether the reads belonged to Gram-negative organisms (with sub-classification of the Negativicutes). The average CpG content per megabase for each bacterial taxonomic group was calculated based on the reads assigned by the taxonomic binning and counting occurrences of “CG” using grep. Taxonomic groups were summarised to species level where strain level identifications were provided.

### Bacterial quantification

Quantitative PCR (qPCR) was performed on the same extracted DNA samples used for the shotgun metagenomic sequencing. qPCR standards were derived from a *Pseudomonas aeruginosa* PAO1 full-length 16S rRNA gene cloned into TOPO TA vector and primers were BAC338F (500 nM) and BAC805R (200 nM) with a BAC516P reporter ((FAM)-TGCCAGCAGCCGCGGTAATAC-(BHQ-1)). The qPCR protocol involved an initial incubation at 95 °C for 10 min, followed by 40 cycles of (95 °C, 15 s; 60 °C, 1 min) with data collected at 60 °C. The reaction was performed using a TaqMan Universal PCR Master Mix kit on a StepOnePlus Real-Time PCR System (Applied Biosystems). Results are shown in Additional file 3.

qPCR provided an absolute 16S rRNA copy number per gram of faeces, and metagenomic sequencing estimated the proportions of different bacteria in the community (with the proportions of binned DNA being corrected for genome length). 16S rRNA copy numbers were sourced for each of these bacterial groups at the greatest possible taxonomic resolution (see Additional file 3) and used to weight the corrected bacterial community proportions (given that bacteria with higher 16S rRNA copy numbers appear to be more numerous by 16S rRNA quantification). The qPCR derived 16S rRNA copies per gram was divided according to these proportions giving the number of 16S rRNA copies for each taxonomic group per gram of faeces. Division of these values by the number of 16S rRNA copies for each bacterial group gave the absolute number of bacteria. Where available, 16S rRNA copy numbers were taken from existing literature [19, 33], and were otherwise calculated from complete NCBI genomes [34]. If no genome was available, the copy number of a higher-ranking taxonomic group was used as an estimate.

### Calculation of immunostimulatory proxies

To estimate the activation of TLR9, the amount of CpG DNA per gram of faeces was calculated using abundance of each taxonomic group and multiplying by the average genome length of that group (see Additional file 3) to give the total amount of DNA for each taxonomic group per gram of faeces. Multiplication by the average CpG copies per megabase of DNA for each group (as previously calculated) then gave an estimate of the total CpG motif content per gram.

The same process was also performed on the DNA motif “GTCGTT”, which has been identified as the optimal motif for stimulation of TLR9 [35]. GTCGTT occurrences per gram were found to be linearly related to CpG per gram and given that data is unavailable for the stimulatory potential of other CpG topologies, CpG per gram was taken forward for this analysis.

To estimate the activation of TLR4, the amount of Gram-negative bacteria in each sample was calculated using the previously derived abundances per taxonomic group. We excluded the order Negativicutes from the total of Gram negative bacteria given the reduced TLR4 stimulation exhibited by *Veillonella parvula* [36], which we extrapolate to be a feature of the family resulting from their genetic distance from other Gram negative organisms.

### Statistics

Clustering of samples by Euclidean distance was performed in MATLAB. Statistical analyses were performed in R studio version 1.2.5042 running R version 4.0.0 and using the Vegan package [37–39]. The CCA used the most abundant taxonomic groups that constitute 95% of the sequencing reads of the dataset. Bacterial counts per gram of faeces were compared using the Wilcoxon Signed-Rank test due to small group sizes. Linear regressions were used to explore the relationship between total bacterial counts and both CpG counts and Gram negative bacteria counts for control infants with the spread of the data being expressed as IQR around the regression line due to sample sizes. Bayesian generalized linear models with binomial distributions, implemented with the arm package [40], were used segregate cases and control groups, with initial models comparing the two sets of cases highlighted in Fig. 4 versus all non-validation control infants. Cases that fell within either NEC group were added or removed iteratively as indicated by the models. The coefficients of factors retained in the two final models were used to calculate predicted probabilities for the validation dataset.

## Supplementary Information

**Additional file 1.** Number of bacteria per gram of faeces for NEC and Control Samples. The number of bacteria calculated per gram of faeces by qPCR for NEC and control samples. X axis shows day of life that the sample was taken, Y axis the number of bacteria per gram of faeces.

**Additional file 2.** Model details. Details of the Bayesian regression models with coefficients determined using our training dataset of 11 control infants and 11 NEC cases. 95% credible intervals have been calculated for each coefficient. The spread of each variable over the training dataset is provided.

**Additional file 3.** Data for sample characterisation. Tab1 – Infant and sample information. Tab 2 – qPCR data use to establish 16S copies per gram of faeces. Tab 3 –Metagenomic assignments with genome lengths, 16S copies per genome and Gram-staining.

**Additional file 4.** Shotgun metagenomic sequencing. Details of shotgun metagenomic sequencing including reads per sample and the accession number relating to the data in the ENA.

## Data Availability

The datasets supporting the conclusions of this article are available in the EBI European Nucleotide Archive repository, https://www.ebi.ac.uk/ena/browser/view/PRJEB15257 and https://www.ebi.ac.uk/ena/browser/view/PRJEB19677, and are included within the article (and its additional file(s)).

## Abbreviations

CCA: Canonical correlation analysis
IRAK-M: Interleukin-1 receptor associated kinase
LPS: Lipopolysaccharide
NEC: Necrotising enterocolitis
NICU: Neonatal intensive care unit
NIHR: National Institute for Health Research
qPCR: Quantitative polymerase chain reaction
TLR: Toll-like receptor
