# Supplementary figures and images for: Premature neonatal gut microbial community patterns supporting an epithelial TLR-mediated pathway for necrotizing enterocolitis
Source: BMC Microbiol. 2021 Aug 6;21:225. doi: 10.1186/s12866-021-02285-0 (PMC8343889; doi:10.1186/s12866-021-02285-0)

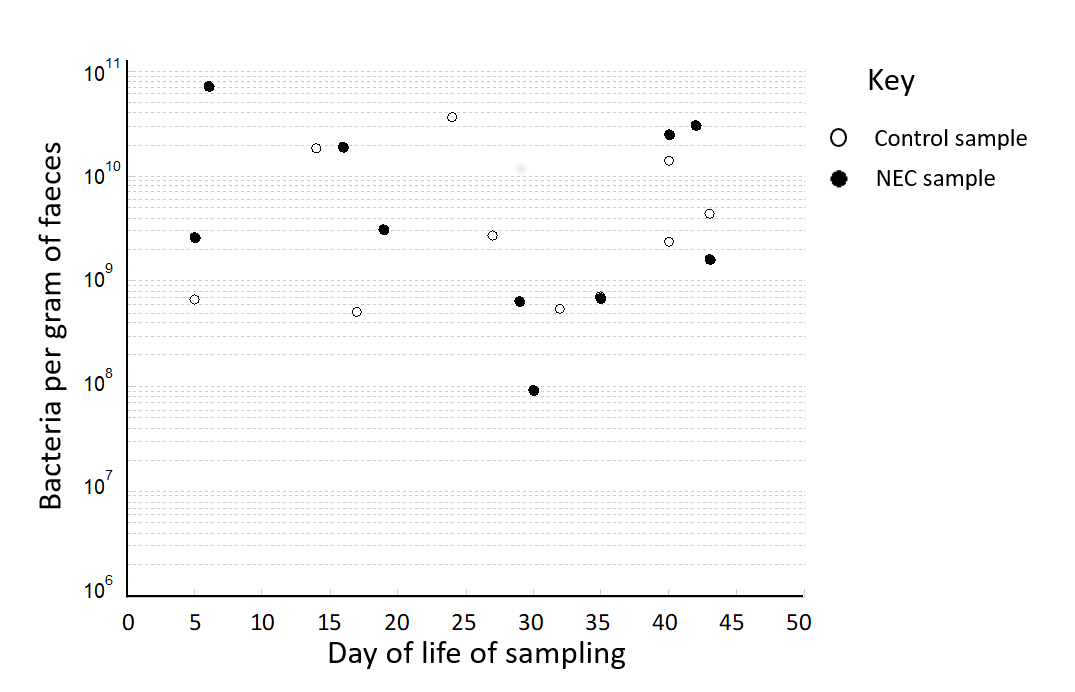

Supplement: Supplementary file 1 — Additional file 1. Number of bacteria per gram of faeces for NEC and Control Samples. The number of bacteria calculated per gram of faeces by qPCR for NEC and control samples. X axis shows day of life that the sample was taken, Y axis the number of bacteria per gram of faeces. [file 12866_2021_2285_MOESM1_ESM.tif]
